# Do dietary practices and household environmental quality mediate socio-economic inequalities in child undernutrition risk in West Africa?

**DOI:** 10.1017/S1368980022002269

**Published:** 2023-05

**Authors:** Duah Dwomoh, Christian Sewor, Samuel K Annim, Saverio Stranges, Ngianga-Bakwin Kandala, A Kofi Amegah

**Affiliations:** 1 Public Health Research Group, Department of Biomedical Sciences, University of Cape Coast, Cape Coast, Ghana; 2 Department of Biostatistics, School of Public Health, University of Ghana, Legon, Accra, Ghana; 3 Department of Applied Economics, School of Economics, University of Cape Coast, Cape Coast, Ghana; 4 Ghana Statistical Service, Accra, Ghana; 5 Department of Epidemiology and Biostatistics and Africa Institute, Western University, London, ON, Canada; 6 Department of Precision Health, Luxembourg Institute of Health, Strassen, Luxembourg; 7 University of Warwick, Division of Health Sciences, Warwick Medical School, Coventry, UK; 8 University of the Witwatersrand, Division of Epidemiology and Biostatistics, School of Public Health, Johannesburg, South Africa

**Keywords:** Maternal education, Household wealth, Undernutrition, IYCF, Household environmental quality, West Africa

## Abstract

**Objective:**

We investigated the relationship between socio-economic status and child undernutrition in West Africa (WA), and further examined the mediating role of dietary practices (measured as minimum dietary diversity (MDD), minimum meal frequency (MMF) and minimum acceptable diet (MAD)) and household environmental quality (HEQ) in the observed relationship.

**Design:**

Thirteen countries were included in the study. We leveraged the most recent Demographic and Health Surveys datasets ranging from 2010 to 2019. Poisson regression model with robust standard errors was used to estimate prevalence ratios and their corresponding 95 % CI. Structural equation modelling was used to conduct the mediation analysis.

**Setting:**

West Africa.

**Participants:**

132 448 under-five children born within 5 years preceding the survey were included.

**Results:**

Overall, 32·5 %, 8·2 %, 20·1 % and 71·7 % of WA children were stunted, wasted, underweight and anaemic, respectively. Prevalence of undernutrition decreased with increasing maternal education and household wealth (Trend *P*-values < 0·001). Secondary or higher maternal education and residence in rich households were associated with statistically significant decrease in the prevalence of stunting, wasting, underweight and anaemia among children in WA. MAD was found to mediate the association of low maternal education and poor household wealth with childhood stunting and underweight by 35·9 % to 44·5 %. MDD, MMF and HEQ did not mediate the observed relationship.

**Conclusions:**

The study findings enables an evaluation and improvement of existing intervention strategies through a socio-economic lens to help address the high burden of child undernutrition in WA and other developing regions.

The prevalence of child undernutrition in low-income and middle-income countries (LMIC) remains unacceptably high^(1)^ with several countries not meeting the Millennium Development Goals target for child undernutrition and unlikely to meet both the 2025 World Health Assembly nutrition targets and 2030 Sustainable Development Goals on child undernutrition^(2)^. This is against the backdrop of the series of interventions implemented worldwide and the huge financial investment coupled with increasing political commitment to address the child undernutrition problem^(2)^.

While global trends reveal a sharp decline in child undernutrition in many parts of the world, regional estimates reveal that this decline occurs at a relatively slower rate in many African countries^(3)^. Within the African region, the highest prevalence of child undernutrition has consistently been observed in the West Africa sub-region^(3,4)^. Child undernutrition is a major determinant of the physical, mental and cognitive development of children and health in later adult life^(5)^. In addition, childhood anaemia, an important determinant of health and development of children is also very prevalent in sub-Saharan Africa (62·3 %) and stands as the leading cause of years lived with disability within the sub-Saharan Africa region^(6,7)^.

These long-term consequences of child undernutrition such as non-communicable diseases in later life, poor physical and cognitive ability and susceptibility to infectious diseases demand an understanding of the dynamics and mechanistic pathways of child nutritional outcomes. It is worth pointing out that, the reason why child undernutrition is still pervasive in the West African region is because of the failure to address the basic and underlying causes in the region. According to a UNICEF framework, the basic and underlying causes of undernutrition consist of environmental, economic and socio-political contextual factors with poverty playing a key role^(8)^. These contextual factors continue to persist in West Africa. For instance, household poverty which is strongly associated with household food insecurity, inadequate healthcare services for both mother and child, and poor household environmental sanitation is pervasive in West Africa in spite of the numerous efforts made to improve the situation^(9)^.

The influence of socio-economic, dietary and environmental factors on child undernutrition is well documented^(4,9–12)^ and affirms the important role these factors play in the high prevalence of child undernutrition in West Africa. However, it is worth noting that these factors influence child undernutrition at varying levels including interacting together to magnify the problem. It is therefore important to disentangle these complexities to help better tailor intervention strategies for addressing the child undernutrition problem. Such an exercise could also help explain the observed variations in child undernutrition levels in West Africa. It is against this background that we set out to understand the pathways through which socio-economic status influences child nutrition to help better evaluate existing policies and interventions for their effectiveness in addressing the high burden of child undernutrition in West Africa and other developing regions.

Leveraging the most recent Demographic and Health Survey (DHS) data, we investigated the relationship between maternal education and household wealth, and child undernutrition in West Africa whilst also examining the mediating role of infant and young child feeding (IYCF) practices and household environmental quality (HEQ) in the observed relationship. We hypothesised that poor socio-economic status would result in poor HEQ through the use of solid fuels for cooking, use of unimproved water sources and unimproved sanitation as well as poor IYCF practices which in turn increases the prevalence of undernutrition (Fig. 1).

**Fig. 1:**
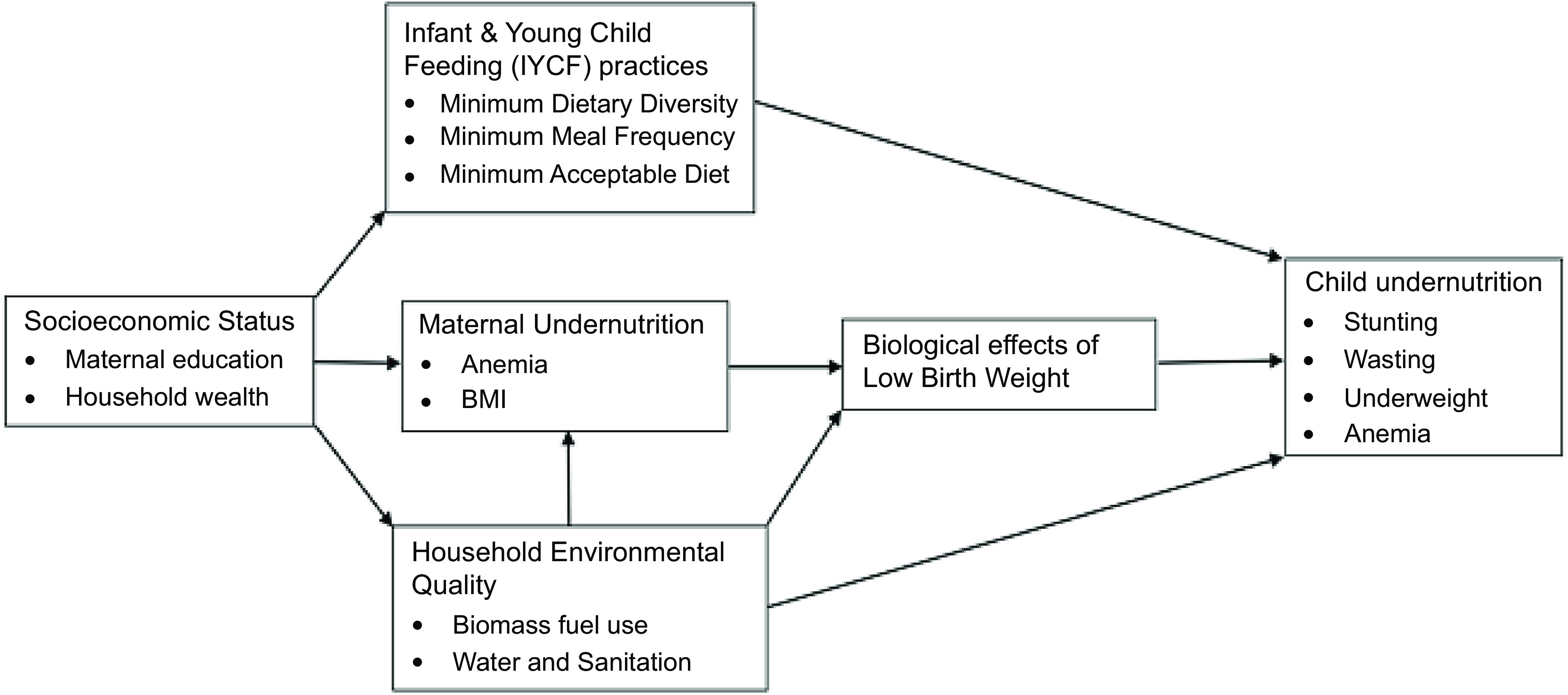
Possible pathways of the association of socio-economic status with child undernutrition

## Methods

We report this study according to the STrengthening the Reporting of OBservational studies in Epidemiology (STROBE) checklist.

### Data source

The study used the most recent DHS dataset for all the included countries. DHS are nationally representative household surveys that collect data on women of reproductive age (15–49 years). The DHS sampling design is based on a two-stage stratified cluster sampling approach. Briefly, the sampling procedure sees the country stratified by region, province or state followed by a random sampling of enumeration areas (EA) within each stratum. The EA are a cluster of households within a well-defined geographical area. The second stage of sampling involves a systematic sampling of approximately 20–30 households within the selected EA. The detailed sampling design for each country-specific DHS can be found in the appendix of the final DHS report available for download at the DHS website (https://dhsprogram.com/).

The data used in this study have one record for every child of a woman interviewed and born within 5 years preceding the survey. The data contains information on the child’s nutritional (stunting, wasting, underweight) and anaemia status, and socio-demographic characteristics of the child, mother and household. The data also have information on postnatal care, immunisation status and other health indicators. The unit of analysis is children of women born in the last 5 years (0–59 months). Characteristics of the study population are presented in Table 1.

**Table 1:** Characteristics of the study population

Country	Year of survey	Estimated population of women aged 15–49 years*	Number of eligible women aged 15–49 years interviewed	Number of under-five children born in the 5 years preceding the survey *n*	%	Number of children alive at the time of the survey *n*	%	Eligible women response rate
Benin	2017	2 635 019	15 928	13 589	9·35	12 651	9·55	98·1
Burkina Faso	2010	3 579 388	17 087	15 044	10·35	13 716	10·36	98·4
Cote d’Ivoire	2011	4 858 181	10 060	7776	5·35	7093	5·36	92·7
Gambia	2013	471 456	10 233	8088	5·56	7788	5·88	90·7
Ghana	2014	6 797 068	9396	5884	4·05	5595	4·22	97·3
Guinea	2018	3 015 710	10 874	7951	5·47	7273	5·49	99·0
Liberia	2019	1 189 170	9239	7606	5·23	5245	3·96	96·4
Mali	2018	4 237 636	10 519	9940	6·84	9275	7·00	97·6
Niger	2012	3 719 064	11 160	12 558	8·64	11 602	8·76	95·4
Nigeria	2018	44911135	41 821	33 924	23·34	30 713	23·19	99·3
Senegal	2019	3 890 229	34 850	6125	4·21	5899	4·45	96·1
Sierra Leone	2019	1 914 452	15 574	9899	6·81	9063	6·84	96·7
Togo	2013	1 677 648	9480	6979	4·80	6535	4·93	97·8
Total		82703757	206 221	143 461		132 448		96·7

%: Unweighted percentages.

* https://www.who.int/data/maternal-newborn-child-adolescent-ageing/indicator-explorer-new/mca/women-of-reproductive-age-(15–49-years)-population-(thousands).

### Study population and sampling size

Thirteen West African countries (Benin, Burkina Faso, Cote d’Ivoire, Ghana, Gambia, Guinea, Liberia, Mali, Niger, Nigeria, Sierra Leone, Senegal and Togo) were included in the study. The DHS survey years ranged from 2010 to 2019. The eligibility criteria for the study were as follows: (1) children aged 0 to 59 months who were born within 5 years preceding the survey; (2) child was alive at the time of the survey and (3) information on the child’s nutritional and anaemia status were available. We included a total of 132 448 children from the thirteen West African countries in the final sample.

### Outcome measures

The outcomes of interest were childhood stunting, wasting, underweight and anaemia. Based on the 2006 WHO child growth standards, children were classified as stunted if their height for age *Z*-score were below – 2 standard deviations (sd) from the median of the reference population. Children were classified as wasted if their weight for height *Z*-scores were below -2 SD from the median of the reference population. Children were classified as underweight if their weight for age *Z*-score were below -2 SD from the median of the reference population. In the DHS survey, these childhood nutritional indicators were computed for children aged 0–59 months. Children aged 6–59 months with Hb levels less than 11·0 g/dl were classified as anaemic.

### Determinant of interest

The determinant of interest was socio-economic status measured by mother’s educational attainment (no education, primary, secondary, or higher) and household wealth (poor, moderate and rich). The DHS programme uses principal component analysis to derive household wealth for each country and categorised it into five levels (poorest, poor, moderate, rich and richest). The IPUMS-DHS contains coded variables on all the indicators including household wealth for all countries participating in the DHS programme and has been standardised to facilitate direct comparison across the countries. In our study, we classified poorest and poor as poor, moderate as moderate, and rich and richest as rich. This re-classification was undertaken to reduce dimension of the covariates to help render the model more parsimonious and facilitate interpretation of the determinant of interest.

### Mediators

The mediators of interest were the following: environmental factors (water source, sanitation and primary cooking fuel) and Infant and Young Child Feeding (IYCF) Practices (minimum dietary diversity, minimum meal frequency (MMF) and minimum acceptable diet (MAD)).

Water sources and sanitation were classified into improved and unimproved sources based on the definition of the WHO/UNICEF Joint Monitoring Programme for Water Supply and Sanitation for the Sustainable Development Goals monitoring period^(13)^. Improved sanitation is defined as shared or non-shared facilities that flush/pour-flush to a piped sewer system, septic tank or pit latrine; ventilated improved pit latrine; pit latrine with a slab and composting toilet or flush elsewhere. Unimproved sanitation includes pit latrine without slab/open pit, bucket toilet, hanging toilet/latrine and others. Improved water sources are the following: piped into dwelling, piped to yard/plot, public tap/standpipe, piped to a neighbour, tube well or borehole, protected well, protected spring, rainwater, tanker truck, cart with a small tank, bottled water and packaged or delivered water. Unimproved water sources are unprotected well, unprotected springs and surface water (river, dam, lake, pond, stream, canal/irrigation channel).

The WHO 2007 indicator for assessing IYCF practices, defines minimum dietary diversity (MDD) as having received four out of seven food groups^(14)^. However, in 2017, WHO changed this definition to five out of eight food groups with a group for breastfeeding added to the groups^(15)^. MDD from 2017 is therefore defined as the proportion of children aged 6–23 months who consumed foods from at least five food groups out of the eight referenced food groups within a 24-h time period (i.e. feeding from five out of eight food groups during the day or night preceding the survey). The eight food groups are the following: (1) breastmilk; (2) grains, roots and tubers; (3) legumes and nuts; (4) dairy products; (5) flesh foods (meat, fish, poultry and liver/organ meats); (6) eggs; (7) vitamin A-rich fruits and vegetables and (8) other fruits and vegetables. We used the WHO 2017 definition for MDD in the present study, i.e. children between the ages of 6 and 23 months who had consumed foods from at least five out of the recommended eight food groups in the last 24 h prior to the survey.

MMF is defined as the proportion of breastfeeding children aged 6–8 months or 9–23 months who received solid or semi-solid or soft foods two and three or more times during the day or night preceding the survey (24 h preceding the interview), respectively. For non-breastfeeding children aged 6–23 months, they must receive solid or semi-solid or soft foods four or more times during the day or night preceding the survey.

MAD is a composite indicator composed of MDD and MMF. For breastfed children, they should meet the MDD and MMF requirement. For non-breastfed children, they should meet the MDD requirement but excluding the dairy products category and MMF, and two or more milk feed.

### Covariates

The following variables were controlled for in the multivariable analysis; child’s age and sex, self-reported birth weight, place of residence (urban *v*. rural), household size, marital status, age at first birth, multiple births, parity, skilled birth attendant at delivery, household ownership of bednet, receipt of vitamin A supplement in the last 6 months, employment status of the mother, diarrhoea in the last 2 weeks preceding survey and child immunisation status.

### Statistical analysis

We pooled data from thirteen West African countries for the analysis. We first quantified the effect of socio-economic status (education and household wealth) on child undernutrition (stunting, wasting, underweight and anaemia) using Poisson regression model with robust standard errors and log link function. Prevalence ratios (PR) and their corresponding 95 % CI were estimated from the models. All the analyses were adjusted for the complex survey design characteristics (sampling weight, clustering and stratification) as provided by DHS. This is to ensure that the effect estimates computed were unbiased and representative of the study population. In the pooled analyses, we re-weighted observations by the country’s population size (i.e. de-normalise the sampling weight) and further adjusted for country and year of survey fixed-effects to account for the unobservable country-level factors and time trend. Marginal effects and their corresponding 95 % CI for interaction between mother’s educational attainment and household wealth status were estimated.

We conducted a causal mediation analysis using generalised structural equation modelling (sem) with Poisson distribution and log link function to investigate the mediating role of IYCF practices (MDD, MMF and MAD) and HEQ in the observed associations conditional on potential pre-exposure covariates as follows: (1) The Poisson regression model that connects the exposures and the mediating variables to outcomes controlling for additional confounding variables is given by
(1)

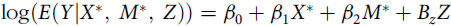




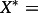
 socio-economic status (household wealth, mothers’ education), 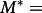 Mediating variables (MDD, MMF, MAD, HEQ) and 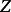 represents other confounding factors and (2) The Poisson regression model that assesses how the exposure influenced the mediator controlling for possible confounding covariates is given by
(2)

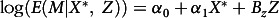




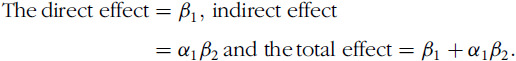




The point estimates and their corresponding standard errors estimated from the model were adjusted for the complex survey design structure (clustering, weighting and stratification).

We used non-linear combination command *(‘nlcom’)* after the Poisson-based generalised sem to generate point estimates and their corresponding standard errors, and *P*-values for the total effect, direct effect and indirect effect. The *‘nlcom’* estimations of the non-linear parameters were based on the delta method. However, for the indirect effect, we relied on bootstrap standard errors. This because *‘nlcom’* computes standard errors using the delta method which assumes that the estimates of the indirect effect are normally distributed. This is, however, not the case as the estimates are usually positively skewed and kurtotic. The *Z*-test and corresponding *P*-values for the indirect effects size estimates would therefore be incorrect with use of *‘nlcom’*. Hence, the recommendation that percentile bootstrap standard errors and confidence intervals be used^(16)^. Since the exposures were categorical variables with more than two levels of the factor, the number of possible tests in the mediation analysis could be large and could inflate Type I error^(16)^. We therefore employed a more consecutive Bonferroni approach for handling the multiple test correction by setting the *P*-value for rejecting the null hypothesis at 0·025 or less which corresponds to a 97·5 % CI.

We generated a latent trait variable (HEQ index) from household sanitation (improved *v*. unimproved), water sources (improved *v*. unimproved) and household cooking fuel (clean *v*. solid) using item response theory with the one-parameter logistic regression model that adjusts for complex survey design characteristics. We used empirical Bayes means to predict the latent HEQ index. Solid fuels include coal/lignite, charcoal, wood, straw/shrub/grass, crops and animal dung. Clean fuels include electricity, liquefied petroleum gas, natural gas and biogas. The HEQ index was categorised into low, medium and high levels based on quantile distribution.

We used Stata MP version 16 (StataCorp., LP) for all the analyses. All statistical tests were two-tailed, and *P* < 0·025 was considered statistically significant.

## Results

Of the 143 461 children born in the 5 years preceding the survey in the thirteen included countries, 132 448 (92·3 %) were alive at the time of the survey and constituted our study sample. The average age (SD) of the children was 1·96 (1·43) years. About 51 % of the children were boys. The proportion of children who lived in rural areas was 64·7 %. About 54 % and 43 % of the mothers interviewed had no education and resided in poor households, respectively.

Table 2 presents the prevalence of child undernutrition in West Africa stratified according to the determinants of interest and mediators. Overall, 32·5 % (95 % CI (31·6, 33·3)) of children were stunted, 8·2 % (95 % CI (7·8, 8·5)) were wasted, 20·1 % (95 % CI (19·5, 20·8)) were underweight and 71·7 % (95 % CI (70·8, 72·6)) were anaemic. Prevalence of undernutrition decreased with increasing maternal education and household wealth status (Trend *P* < 0·001). The prevalence of undernutrition was higher in households with unimproved water sources and sanitation and lower in households using clean fuels for cooking.

**Table 2:** Prevalence of child undernutrition by exposure and mediation factors among children under-five in West Africa

	Stunting %	95 % CI	Wasting %	95 % CI	Underweight %	95 % CI	Anaemia %	95 % CI
Overall prevalence	32·46	31·60, 33·34	8·15	7·81, 8·50	20·12	19·46, 20·80	71·69	70·79, 72·58
Mother’s education								
None	40·24	39·17, 41·33	10·14	9·67, 10·64	26·09	25·21, 26·98	77·53	76·50, 78·53
Primary	31·40	29·92, 32·91	6·72	5·99, 7·54	17·09	15·97, 18·27	72·48	70·63, 74·26
Secondary+	19·43	18·34, 20·57	5·40	4·90, 5·96	11·27	10·45, 12·15	61·75	60·11, 63·36
Wealth status								
Poor	42·23	40·94, 43·53	9·61	9·07, 10·18	26·72	25·63, 27·83	78·93	77·88, 79·94
Moderate	33·26	31·89, 34·65	7·80	7·13, 8·52	19·19	18·10, 20·33	71·71	69·99, 73·37
Rich	21·65	20·60, 22·75	6·78	6·30, 7·30	13·59	12·80, 14·42	64·05	62·59, 65·48
Water source								
Improved	29·19	28·26, 30·14	7·91	7·52, 8·32	18·35	17·62, 19·10	69·65	68·60, 70·67
Unimproved	41·46	39·97, 42·96	8·96	8·34, 9·62	25·01	23·78, 26·28	77·19	75·75, 78·57
Sanitation								
Improved	26·26	25·14, 27·40	6·70	6·28, 7·14	15·57	14·78, 16·39	65·57	64·29, 66·83
Unimproved	38·45	37·39, 39·52	9·59	9·10, 10·11	24·49	23·59, 25·41	77·42	76·45, 78·36
Type of cooking fuel								
Solid fuel	34·29	33·41, 35·18	8·53	8·16, 8·90	21·23	20·54, 21·93	73·63	72·77, 74·48
Clean fuel	13·73	11·68, 16·07	4·53	3·57, 5·72	8·79	7·16, 10·75	51·28	47·88, 54·67
Household environmental quality								
Low	41·78	40·19, 43·39	9·35	8·64, 10·11	25·81	24·50, 27·17	77·78	76·20, 79·29
Medium	36·77	35·63, 37·93	9·29	8·71, 9·90	23·32	22·33, 24·34	76·89	75·81, 77·94
High	24·02	22·91, 25·17	6·54	6·10, 7·00	14·45	13·65, 15·28	64·11	62·74, 65·46
Minimum dietary diversity								
Inadequate	35·23	34·27, 36·20	7·89	7·51, 8·23	21·29	20·54, 22·05	71·58	70·65, 72·49
Adequate	26·16	24·15, 28·27	8·78	7·56, 10·17	16·70	15·08, 18·46	72·95	70·60, 75·18
Minimum meal frequency								
Inadequate	35·67	34·65, 36·70	7·10	6·73, 7·50	20·85	20·08, 21·65	69·67	68·69, 70·63
Adequate	29·15	27·72, 30·62	11·59	10·71, 12·54	20·94	19·77, 22·15	80·00	78·59, 81·34
Minimum acceptable diet								
Inadequate	35·06	34·10, 36·03	7·91	7·54, 8·30	21·27	20·52, 22·03	71·45	70·54, 72·35
Adequate	23·86	21·61, 26·27	8·94	7·34, 10·84	14·46	12·72, 16·39	75·66	72·79, 78·31

Table 3 presents adjusted PR estimated from modified Poisson regression for child undernutrition according to mother’s educational attainment and household wealth, independently and jointly. Secondary or higher education level of mother was associated with 33 % (adjusted PR (aPR) = 0·67, 95 % CI (0·61, 0·75), 44 % (aPR = 0·56, 95 % CI (0·43, 0·73)), 47 % (aPR = 0·53, 95 % CI (0·46, 0·62)) and 9 % (aPR = 0·91, 95 % CI (0·84, 0·98)) decrease in the prevalence of stunting, wasting, underweight and anaemia, respectively, among West African children compared to no formal education. Residence in households classified as rich was associated with 21 % (aPR = 0·79, 95 % CI (0·72, 0·87)), 19 % (aPR = 0·81, 95 % CI (0·66, 0·99)), 23 % (aPR = 0·77, 95 % CI (0·68, 0·88)) and 10 % (aPR = 0·90, 95 % CI (0·84, 0·96)) decrease in the prevalence of stunting, wasting, underweight and anaemia, respectively, among children in West Africa compared to residence in poor households.

**Table 3:** Prevalence ratios (PR) estimated from modified Poisson regression for child undernutrition according to mother’s educational attainment and household wealth status

Determinant of interest	Stunting aPR	95 % CI	Wasting aPR	95 % CI	Underweight aPR	95 % CI	Anaemia aPR	95 % CI
Mother’s education								
None	1·00		1·00		1·00		1·00	
Primary	0·87	0·80, 0·94	0·67	0·54, 0·84	0·74	0·65, 0·83	1·00	0·94, 1·06
Secondary or higher	0·67	0·61, 0·75	0·56	0·43, 0·73	0·53	0·46, 0·62	0·91	0·84, 0·98
Trend *P*-value	*P* < 0·001		*P* < 0·001		*P* < 0·001		*P* < 0·05	
Household wealth								
Poor	1·00		1·00		1·00		1·00	
Moderate	0·90	0·83, 0·97	0·94	0·79, 1·12	0·81	0·73, 0·90	1·02	0·97, 1·08
Rich	0·79	0·72, 0·87	0·81	0·66, 0·99	0·77	0·68, 0·88	0·90	0·84, 0·96
Trend *P*-value	*P* < 0·001		*P* < 0·001		*P* < 0·001		*P* < 0·05	

aPR: adjusted prevalence ratio.

**Fig. 2:**
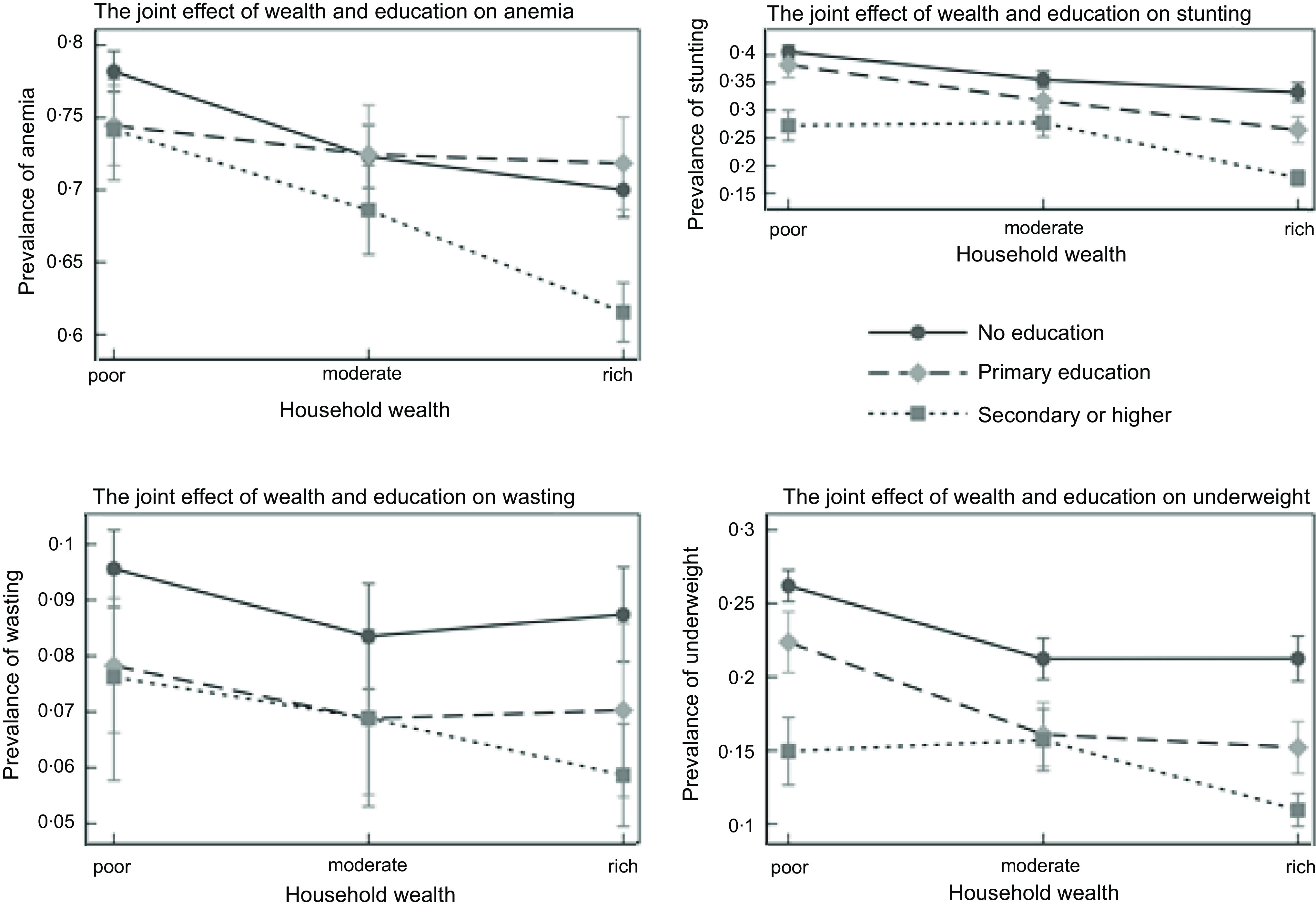
Plot of the marginal effects estimated from the interaction of mother’s educational attainment and household wealth status on child undernutrition in West Africa

Table 4 presents marginal effects (predicted prevalence) estimated from the interaction between mother’s educational attainment and household wealth status. Prevalence of stunting, wasting, underweight and anaemia was highest among children whose mothers had no education and resided in poor households compared to children whose mothers had secondary or higher education and resided in rich households. However, the prevalence of stunting, wasting and underweight was higher among children whose mothers were not educated but resided in rich households compared to children whose mothers had secondary or higher education but resided in poor households. Figure 2 is a plot of the marginal effects estimated from the interaction of mother’s educational attainment and household wealth status on child undernutrition in West Africa.

**Table 4:** Marginal effects (predicted prevalence) estimated from interaction of mother’s educational attainment and household wealth status on child undernutrition in West Africa

	Predicted prevalence of stunting %	95 % CI	Predicted prevalence of wasting %	95 % CI	Predicted prevalence of underweight %	95 % CI	Predicted prevalence of anaemia %	95 % CI
No education and poor wealth	44·86	42·91, 46·80	12·59	11·33, 13·86	32·42	30·62, 34·22	60·87	58·66, 63·09
Primary education and poor wealth	41·35	37·25, 45·46	9·35	6·72, 11·97	25·79	22·01, 29·58	60·08	55·61, 64·56
Secondary education and poor wealth	31·03	25·50, 36·56	8·42	4·59, 12·27	14·03	9·74, 18·32	49·24	43·03, 55·46
No education and moderate wealth	39·57	36·61, 42·53	11·43	9·30, 13·56	25·63	22·75, 28·51	61·70	58·28, 65·12
Primary education and moderate wealth	34·75	29·33, 40·17	8·70	5·36, 12·04	17·67	13·40, 21·94	61·07	55·27, 66·87
Secondary education and moderate wealth	29·63	24·64, 34·62	7·84	4·76, 10·92	17·38	13·34, 21·42	56·57	51·15, 62·02
No education and rich	38·13	34·89, 41·36	11·80	4·76, 10·92	25·94	22·82, 29·06	51·87	47·88, 55·86
Primary education and rich	28·98	23·75, 34·21	5·46	3·54, 7·38	17·78	13·23, 22·34	54·64	47·99, 61·30
Secondary education and rich	22·84	19·67, 26·02	5·05	3·42, 6·68	12·95	10·64, 15·25	50·34	46·11, 54·59

Table 5 presents the mediation effect of dietary and environmental factors in the association of mother’s educational attainment and household wealth status with child undernutrition in West Africa. MAD was found to mediate 37 % (95 % CI (11·5, 61·5)) and 44·5 % (95 % CI (24·1, 65·1)) of the observed relationships between household wealth, and stunting and underweight, respectively. MAD mediated 35·9 % (95 % CI (12·4, 59·3)) and 39·2 % (95 % CI (20·5, 58·0)) of the observed relationships between mother’s education, and stunting and underweight, respectively. MAD was found not to be a mediator of the observed relationships between household wealth and mother’s education, and childhood wasting and anaemia. MMF, MDD and HEQ were also found not to be a mediator of the observed associations of mother’s education and household wealth with all the outcomes studied.

**Table 5:** Mediation effect of dietary and environmental factors in the association of mother’s educational attainment and wealth status with child undernutrition in West Africa

	Stunting		Underweight	
	aPR	95 % CI	aPR	95 % CI
**Determinant: household wealth** **Mediator: minimum acceptable diet (MAD)**				
Total indirect effect	0·83	0·69, 0·98*	0·70	0·54, 0·90**
Total direct effect	0·72	0·63, 0·83***	0·64	0·53, 0·77***
Total effect	0·60	0·48, 0·76***	0·45	0·33, 0·62***
	MAD %	95 % CI	MAD %	95 % CI
Percentage of total effect of household wealth that is mediated by MAD %	37	11·5, 61·5***	44·5	24·1, 65·1***
**Determinant: mother’s education** **Mediator: minimum acceptable diet (MAD)**				
	aPR	95 % CI	aPR	95 % CI
Total indirect effect	0·74	0·55, 0·99*	0·56	0·38, 0·84**
Total direct effect	0·58	0·51, 0·67***	0·41	0·33, 0·51***
Total effect	0·43	0·31, 0·60***	0·23	0·15, 0·36***
	MAD %	95 % CI	MAD %	95 % CI
Percentage of total effect of mothers education that is mediated by MAD % (95 % CI)	35·9	12·4, 59·3**	39·2	20·5, 58·0**

aPR: adjusted prevalence ratio from the modified Poisson regression model.

* *P* < 0·05.

** *P* < 0·01.

*** *P* < 0·001.

Note: The following variables were accounted for in the multivariable models for the mediators and undernutrition indicators outcomes: child’s age, sex, perceived birth weight, place of residence (urban, rural), household size, marital status, age at first birth, multiple births, child immunisation status and whether the mother is currently working, country and year fixed effect.

## Discussion

Overall, 32·5 %, 8·2 %, 20·1 % and 71·7 % of West African children were stunted, wasted, underweight and anaemic, respectively. Prevalence of undernutrition decreased with increasing maternal education and household wealth (Trend *P* < 0·001). Secondary/higher maternal education and residence in rich households were associated with decreased prevalence of stunting, wasting, underweight and anaemia. MAD mediated the observed relationships between household wealth and mother’s education, and stunting and underweight. The percentage mediation ranged from 35·9 % to 44·5 %. MMF, MDD and HEQ were found not to be mediators of the observed associations.

### Synthesis with previous evidence

The prevalence estimates from our study were higher than recent regional estimates for stunting (27·0 %)^(17)^, wasting (7·5 %)^(17)^ and underweight (19·2 %)^(3)^, but lower for anaemia (75 %)^(18)^. This observation is not surprising owing to the pervasiveness of the basic and underlying factors that drives child undernutrition such as environmental, economic and socio-political factors in West Africa. We found prevalence of child undernutrition to be highest among children whose mothers’ had low educational attainment, poor wealth status, and reside in households with poor environmental conditions (i.e. unimproved water source, unimproved sanitation, use of solid fuels for cooking). Several studies have documented child undernutrition to be very prevalent in poor households^(8,19,20)^. Barros *et al*.^(19)^ observed a sharp reduction in undernutrition levels among children in the upper wealth quintile compared to the other four wealth quintiles in the African region. This observation was recently corroborated in a multi-country study conducted in thirty-five sub-Saharan African countries^(21)^. This study found childhood stunting, underweight, wasting and anaemia to be less prevalent in households with higher wealth status compared to households with lower household wealth status^(21)^. Woldie *et al*.^(20)^ also observed in their study conducted in Northeast Ethiopia that, children born to families in the lowest wealth quintiles were three times more likely to be anaemic than children born to families in the highest wealth quintile.

This increased prevalence of child undernutrition often observed among poor households can be attributed to a number of factors. Studies have suggested that poor households often have less purchasing power which limits their ability to purchase diversified and nutrient-rich foods^(20)^ and accounts for the wide disparities in the consumption of nutrient-rich foods often observed among children in poor and rich households^(8)^. Also, poor household wealth is associated with household food insecurity^(22)^ which is a well-documented risk factor for child undernutrition through macro- and micro-nutrient deficiencies^(8,19,23,24)^. Also, children born in rich households are often more likely to have access to comprehensive neonatal health services including all the immunisation schedules and nutrient supplementation which are important for proper child growth and development^(19,25)^. Children born in poor households are also at increased risk of being exposed to poor environmental conditions such as unsafe water, poor sanitation and household air pollution from the use of solid fuels for cooking, which have also been identified as important risk factors for child undernutrition^(19,26,27)^.

Our findings on the association of low maternal education attainment with child stunting, underweight, wasting and anaemia corroborates the findings of previous studies^(10,11,18,28–30)^. High maternal education attainment confers better nutritional status of children through proper child welfare and feeding practices, household environmental condition and participation of mothers in the household decision-making process. Educated mothers are often very conscious of the health of their children and as a result, are more likely to adopt scientifically proven feeding practices as it would help to improve their children’s nutritional status^(20)^. According to Barros *et al.* ^(19)^, educated mothers are more likely to contribute to the family’s income thereby enabling them to actively participate in family decisions particularly as it relates to the health and welfare of their children. Boah *et al*.^(31)^ also found children born to mothers with high autonomy to be at reduced odds of stunting. Our findings on the mediating role of IYCF practices and HEQ in the observed association reinforces this position. Minimum dietary diversity, minimum meal frequency and MAD have been associated with child undernutrition^(32)^. Poor water, sanitation and hygiene practices have also been associated with increased incidence of waterborne diseases among children, especially diarrhoea, which could potentially impair food absorption leading to micronutrient deficiencies and consequently, childhood anaemia and stunting^(33)^. To the best of our knowledge this study is the first to examine the mediating role of IYCF practices and HEQ in the association of SES (maternal education, household wealth) with child undernutrition.

Prevalence of wasting and anaemia were found to be high among children who had adequate nutrition as per the IYCF indicators. The WHO IYCF indicators are valuable tools for broadly assessing diet quality of children in developing countries to inform the development of sound interventions for addressing child undernutrition in these countries. However, concerns have been raised as to whether the IYCF indicators are very effective parameters for identifying populations at risk of undernutrition. The findings of our study possibly add to these concerns raised. A study conducted in Northern Ghana^(34)^ for instance found levels of wasting and underweight to increase despite improvements in the prevalence of core IYCF indicators and does corroborates the findings of our study. A study that synthesised the evidence on the association of IYCF indicators with child anthropometric indicators^(35)^ also found the associations to be mixed across countries. The authors concluded that the lack of sensitivity and specificity of the IYCF indicators may contribute to the inconsistent associations observed in their study and called for additional measures of diet quality and quantity to help understand how specific IYCF practices relate to child growth faltering. A study conducted in rural Cambodia concluded that child feeding index that incorporates several nutrition information is superior to the WHO IYCF indicators and are needed to understand the association between appropriate infant feeding practices and child growth^(36)^. Besides, child undernutrition is influenced by contextual factors and could also explain the observations of our study. Reviews conducted by Obasohan and colleagues^(37,38)^ noted that child feeding practices although important were among the least reported consistent risk factors of anaemia and undernutrition in comparison to contextual factors such as child age and sex, maternal age and education, household wealth and place of residence. Also, DHS surveys collect information on dietary practices within the last 2 weeks prior to the survey with the information collected not likely to accurately represent diet history of the children assessed. Since child undernutrition are chronic conditions which take a long duration to develop, recent dietary practices as captured in DHS surveys may not be reflective of children’s long-term dietary practices. We do not also rule out reverse causation owing to the cross-sectional design applied. Anaemic and wasted children may have higher meal frequency to compensate for their poor nutritional status.

In the interaction analysis, the highest prevalence of child undernutrition was observed among mothers with low educational attainment and residing in poor households. Also, the lowest prevalence of child undernutrition was observed among highly educated mothers who reside in rich households. This finding confirms the critical role of maternal education and household wealth in improving child nutrition. To date, only three studies^(39–41)^ have examined the interaction between maternal education and household wealth with child nutrition indicators. Leroy *et al*.^(39)^ found a statistically significant interaction between maternal schooling and household wealth on childhood stunting. Ruel and Menon^(40)^ observed a significant two-way interactive effect between child feeding practices and various socio-economic status variables such as maternal ethnicity, maternal education and household wealth on childhood stunting. Makoka^(41)^ on the other hand found no statistically significant interaction between maternal education and household wealth on stunting and wasting. Educated mothers are noted to be more effective than their uneducated counterparts at using the household income to improve the health of children in the household^(42)^.

MAD mediated the observed relationship between mother’s education and household wealth status, and stunting and underweight. MDD and MMF were found not to be mediators of the observed relationships and could be attributed to the fact that, MAD which combines both MDD and MMF, is more applicable across all socio-cultural contexts. For instance, receipt of solid or semi-solid or soft foods under MMF could mean different food items in different socio-cultural settings. With MAD combining both MDD and MMF, it is possible the combined benefits derived from both indicators may have led to the mediation effect observed for this indicator.

### Validity issues

The use of DHS data to test our hypothesis has several advantages owing to the high response rates of DHS surveys and increased statistical power derived from the large sample sizes. DHS surveys rely on a standardised data collection tool and adequately trained interviewers across countries and assures data quality in all the included countries. Also, DHS data are nationally representative through the use of a two-stage stratified cluster sampling approach for obtaining the sample. Selection bias is therefore not a problem in DHS surveys and assures generalisability of the study findings to the entire West Africa region. The potential for outcome misclassification is also minimised in DHS surveys owing to the use of a standardised protocol and well-calibrated instruments across all countries for measuring health outcomes including the child nutritional outcomes studied.

It is worth pointing out the advantage of fitting a modified Poisson regression with robust SE for estimating prevalence ratios (PR) for binary outcomes in cross-sectional studies as against odds ratios (OR) estimated from binary logistic regression. Firstly, OR overestimate PR, the effect measure of choice in cross-sectional studies, especially when the outcomes are common^(43,44)^ as in our study. Secondly, confounding and interaction are dependent on the measure of effect and as a result, controlling for confounding in OR estimation is not the same as PR estimation^(45,46)^. Therefore, according to Barros and Hirakata^(41)^, interpreting the OR as if it were a PR is inadequate not only in terms of the possible overestimation but also because confounding may not be appropriately controlled.

The advantages of using structural equation modelling (sem) for causal mediation analysis have been well documented^(47)^. Paramount among these advantages is the fact that sem is designed to test more complicated mediation models in a single analysis as was the case in this study based on our conceptual framework. Also, use of sem approach allows for simplifying testing of the mediation hypotheses in the presence of multiple independent variables and ease of interpretation of the estimation and results^(47)^.

The study, however, has a few shortcomings. Firstly, besides the covariates that were adjusted for in the multivariable analysis, there are other individual (i.e. maternal health-seeking behaviour) and country-level unobserved factors (i.e. health system access and services) that could potentially confound the relationship studied. These covariates are not measured in the DHS surveys and hence the potential for confounding bias in the study cannot be ruled out. Secondly, the cross-sectional data used does not allow temporality to be established.

In conducting mediation analysis, it has been suggested that, the mediator must significantly influence the outcome measure in the model equation that contains the independent variable and mediator^(48)^. We could not therefore present the mediation effect of MAD in the observed relationship between mother’s education and household wealth, and anaemia and wasting owing to the mediators not having a statistically significant effect on these two outcomes. For the same reason, the mediation effect of MMF, MDD and HEQ in the observed association of mother’s education and household wealth status with all the studied outcomes were also not presented. The mediation effect of HEQ in the observed relationship between household wealth on child undernutrition was not assessed. This is because the environmental variables used in developing the HEQ index are included in the derivation of wealth status by the DHS surveys. Assessing their mediation effect will thus produce bias.

The IYCF indicators only pertain to children aged 6 to 23 months and hence the mediation analysis was restricted to this age group and not the entire study sample.

## Conclusion

The prevalence of child undernutrition was found to be high in the West Africa region. We also found higher maternal education and household wealth to be associated with decreased prevalence of childhood stunting, wasting, underweight and anaemia in West Africa with IYCF practices mediating the observed associations. The findings of the study suggest that in order to address socio-economic inequalities in child undernutrition and anaemia in West Africa there is the need for governments to implement programmes that improves maternal literacy and household income. Such interventions are likely to translate into improved child feeding practices. The study findings also provide the evidence base for evaluating existing intervention strategies for effectiveness through a socio-economic lens if we are to address the high burden of child undernutrition in West Africa and other developing regions of the world.
